# The Impact of Rumen-Protected L-Arginine Oral Supplementation on Libido, Semen Quality, Reproductive Organ Biometry, and Serum Biochemical Parameters of Rams

**DOI:** 10.3389/fvets.2022.899434

**Published:** 2022-06-24

**Authors:** Hassan A. Hussein, Ahmed S. A. Hassaneen, Montaser E. Ali, Ramya A. Sindi, Abdallah M. Ashour, Salem M. Fahmy, Ayman A. Swelum, Ahmed E. Ahmed

**Affiliations:** ^1^Department of Theriogenology, Faculty of Veterinary Medicine, Assiut University, Assiut, Egypt; ^2^Department of Theriogenology, Obstetrics, and Artificial Insemination, Faculty of Veterinary Medicine, South Valley University, Qena, Egypt; ^3^Department of Animal Production, Faculty of Agriculture, Al-Azhar University “Assiut Branch”, Assiut, Egypt; ^4^Department of Laboratory Medicine, Faculty of Applied Medical Sciences, Umm Al-Qura University, Mecca, Saudi Arabia; ^5^Department of Animal Production, Faculty of Agriculture, Al-Azhar University, Cairo, Egypt; ^6^Department of Theriogenology, Faculty of Veterinary Medicine, Zagazig University, Zagazig, Egypt; ^7^Department of Biology, College of Science, King Khalid University, Abha, Saudi Arabia; ^8^Department of Theriogenology, Faculty of Veterinary Medicine, South Valley University, Qena, Egypt

**Keywords:** glucose, pelvic genitalia, pen libido test, ram genital system, ultrasonography

## Abstract

This study aimed to investigate the effect of oral supplementation of rumen-protected L-arginine on semen quality, testes, and accessory genital glands biometry in rams. Ten apparently healthy and fertile rams were randomly divided into two equal groups; control, and rumen-protected L-arginine (20 mg/Kg body weight for 30 days) treated group. In all rams, ultrasonographic measurements of the testes and the accessory genital glands and blood sampling were performed at day (D)10, D20, and D30 (D0 is the start of supplementation). Semen ejaculates were collected twice/week and semen quantity, and quality was examined. Our results showed that, in the L-arginine treated group, there were significant increase in the ultrasound biometric measurement of right seminal vesicle (RSV) and right Cowper's gland (RCG) at D10, both testes, tail of the epididymis (TE), SV, and CG of both sides at D20, and of both testes, RTE, RSV, RCG, and LSV at D30. Semen quality and quantity parameters were significantly improved in L-arginine treated group. Moreover, testosterone level in the L-arginine treated group was significantly higher than that in the Control group. Serum thyroxine and glutathione peroxidase concentrations were significantly higher in the L-arginine treated group. The present study concluded that oral supplementation with rumen-protected L-arginine is beneficial in improvement of rams' fertility.

## Introduction

Basically, in ruminants, protein is predominately degraded in the rumen; therefore, specific amino acids (AAs), such as arginine, fail to reach the small intestine at similar concentrations to the feed. Technologies are being developed to avoid ruminal degradation and allow AAs to reach the intestine without affecting their digestibility. Mateo et al. reported that arginine supplementation may reduce AAs degradation. L-arginine is catabolized to ornithine and urea by arginase in the body. L-arginine is involved in synthesis of chemical substances, such as proteins, glucose, glycogen, nitric oxide (NO), ornithine, and urea (1). Moreover, it is involved in the biosynthesis of creatine, glutamate, and polyamines, as well as some hormones, such as insulin, prolactin, glucagon, and growth hormone (2).

Polyamines (putrescine, spermine, and spermidine) are important biomolecules for cell growth and differentiation that are found in the seminal plasma of ram spermatozoa and other parts of the ram reproductive system, especially the epididymis (3). Since more than a half century, it was reported that spermatozoa motility increased in the infertile individuals who were given 0.5 g/day L-arginine orally for about 2 months (4).

It should be noted that the arginine increase the synthesis and release of NO and the testicular blood flow, which improves performance of the testis in production of testosterone hormone (5, 6). Moreover, Yasuda reported that NO concentrations are increased by increasing the release of cyclic guanosine monophosphate, which is a vasodilator that increases the testicular blood flow (6). The NO-cyclic guanosine monophosphate (NO-cGMP) pathway has been found to play a major role in male sexual function (7).

Energy is an essential requirement for male reproductive performance. The reproductive fertility of males is highly dependent of glucose uptake and metabolism by testicular tissue. Glucose metabolism is critical for normal functions of the testicular cells and more specifically, spermatogenesis (8). In addition, the thyroid hormones (THs) play an important role in the utilization of energy, oxygen consumption, and overall metabolism (9). Only a very small fraction of the total circulating THs is biologically available as the two free forms; free tri-iodothyronine (T3) and free thyroxine (T4) that are accessible to the tissues (10). Thyroxine is considered the precursor for T3, Furthermore, T3 at the ram testes stimulates androgen release in the Leydig cells (11). Moreover, T3 plays an important role in the differentiation of the seminiferous epithelium, and in rodents, it was reported that T3 has a synergistic effect with luteinizing hormone in maturation of Leydig (12).

There is a paucity of the available data on the effect of AAs, such as arginine, on the semen characteristics and the male fertility with contradictory results; some studies previously reported that supplementation of arginine improves concentration, and motility of spermatozoa (5, 13), while other studies did not observe any improvement in semen characteristics or fertility rate (14, 15).

The present study has been designed to evaluate the impact of oral supplementation of rumen-protected L-arg, as an easily applicable route of supplementation, on the ram fertility, such as the testes and accessory glands biometry, semen qualitative and quantitative parameters, testosterone concentration, and the related biochemical and hormonal parameters.

## Materials and Methods

The experiment was conducted during the period of the high-breeding season from September to November when the lowest (minimum) and high (maximum, °C) temperature ranged between 22–36°C and 12–27°C, respectively.

### Ethical Approval

All experimental proceedings in this study were in accordance with the Egyptian Medical Research Ethics Committee (no. 14-126) and in accordance with the Ethics Committee on Animal Experimentation of Al-Azaher University, Faculty of Agriculture.

### Animals and Experimental Design

A total number of 10 apparently healthy, fertile Ossimirams with a bodyweight of 49–54 Kg and aged 2–3 years were included in this study. Rams were housed in sheds during the experimental periods under natural daylight and fed on daily farm ration, which formulated according to the requirement for mature ram according to National Research Council (NRC; 1985) for sheep and consists of 25% wheat straw and 75% concentrate mixture. Animals had a free access to water ad libitum.

### Study Location

This study was conducted at the Animal Production Farm of Faculty of Agriculture, Al-Azaher University (Assuit branch) which is located at 70 m above mean sea level, latitude 27.18° N, and longitude 31.19° E.

### Experimental Design and Formulation of Bypass L-Arginine Pellets

**Figure 1:**
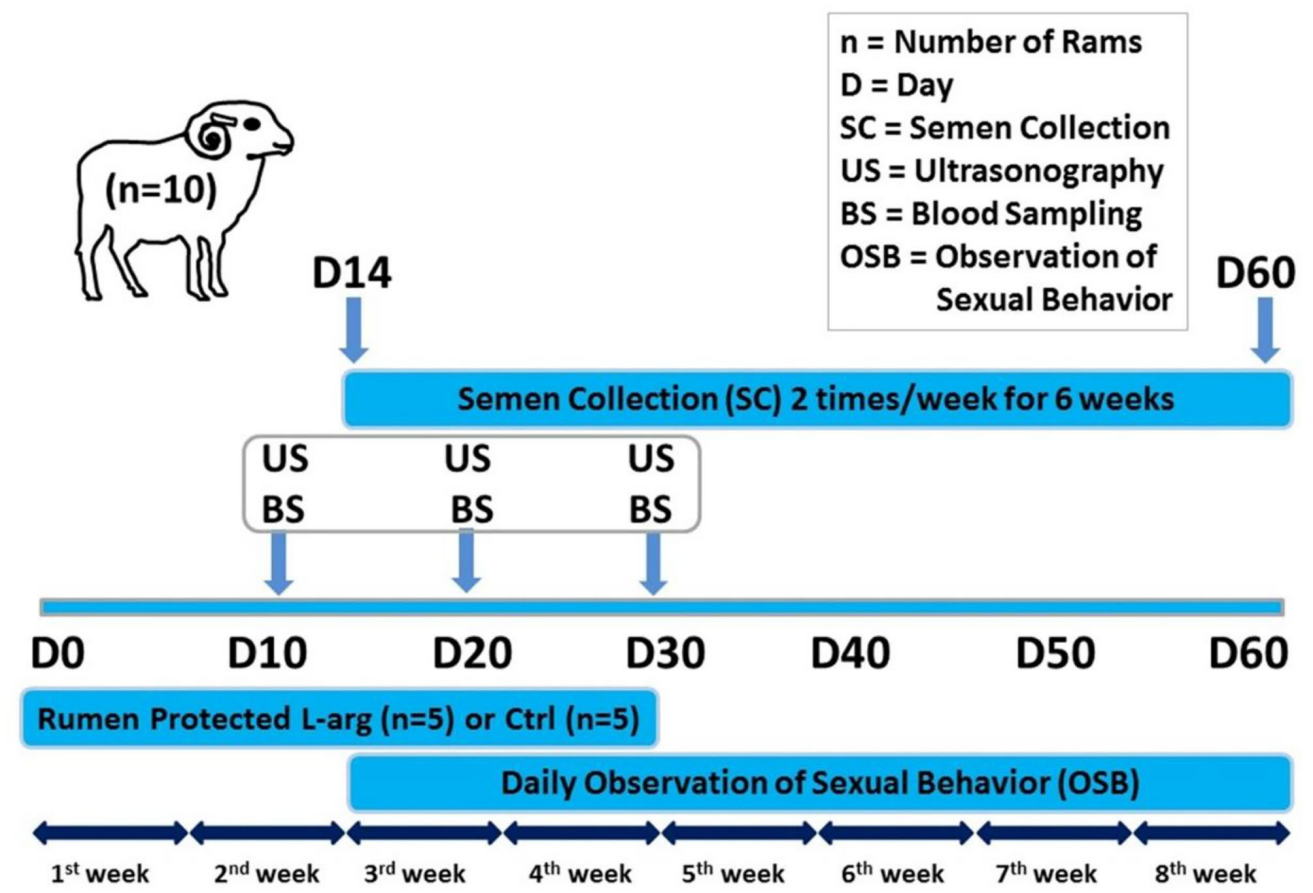
Schematic diagram showing the experimental protocol for the effect of oral administration of rumen-protected L-arginine on the testes and accessory genital glands of rams. Rumen-protected L-arginine was supplemented for 30 days (D0: day of the start of supplementation). Ultrasonography and blood sampling for further biochemical and hormonal assays were performed on D10, D20, and D30. Ram sexual behavior was daily observed and collection of ram semen (2 times/week) was done starting from D14 until D60.

The rams were randomly divided into two equal groups; the control group, and rumen-protected L-arginine treated group 20 mg/Kg body weight (L-arginine pure, 25 gm, C6H14N4O2) for 30 days (Figure 1) (16).

L-arginine was modified to avoid its degradation in the rumen by two different layers (16); the first layer consisted of L-arginine and barium sulfate as a non-functional polymer, briefly, L-arginine was added to the cellulose acetate phthalate (1:1) and then mixed well, after that, the barium sulfate was added to the previous mixture. Barium sulfate provides density to the L-arginine in form of pellets to prevent it from being damaged during rumination; the other outer layer consisted of a functional polymer prepared by ether solution with the capacity to dissolve at pH above 7.2, which enables the pellets to release of L-arginine in the intestine. The ratio between the L-arg and the polymer in the pellets was 4:1, and the amount of L-arg in the pellets was evaluated as previously reported (17).

### Ultrasonographic Examination of the Testes and Accessory Genital Glands

**Figure 2:**
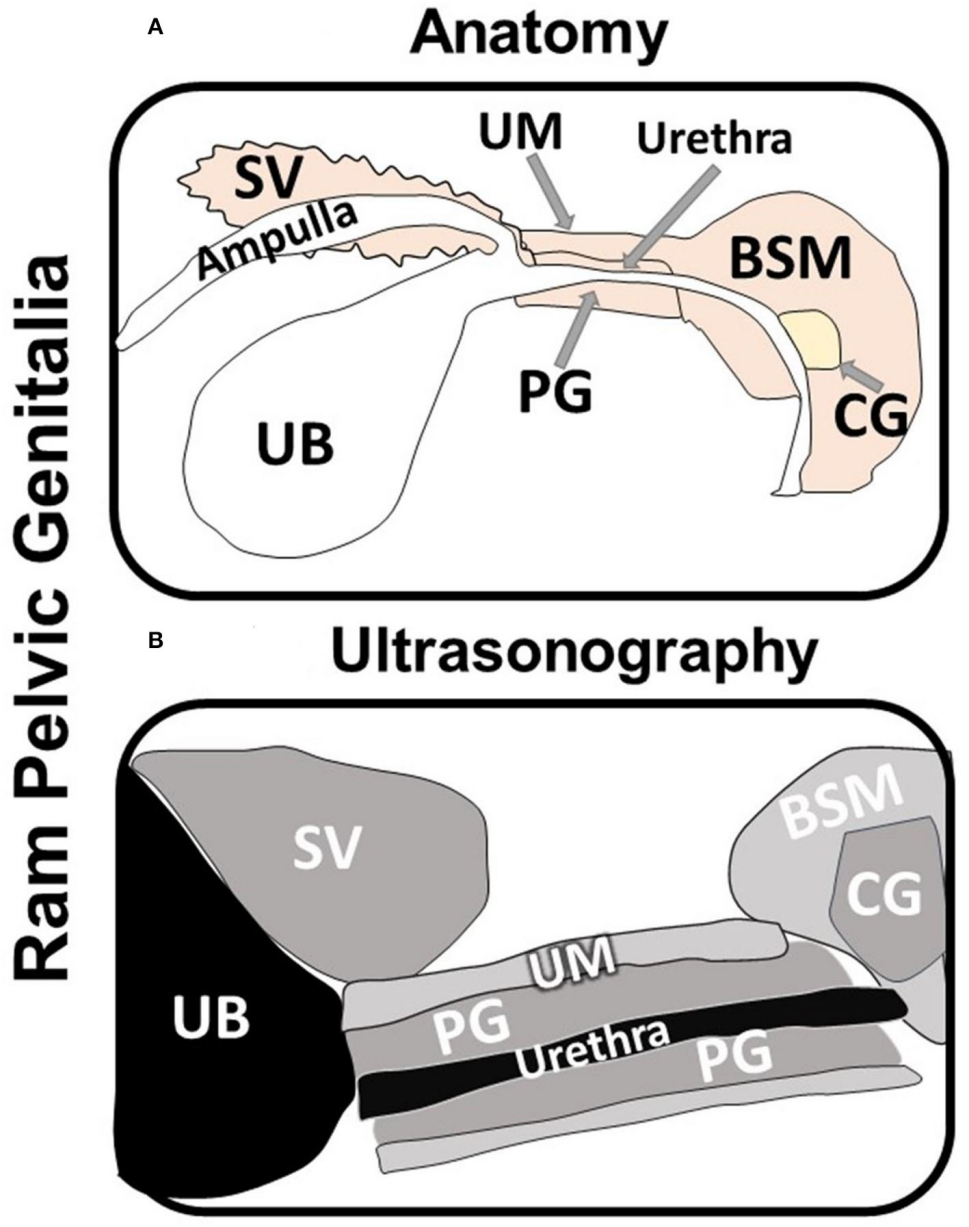
Schematic diagram showing the anatomy **(A)** and the ultrasonography **(B)** of the ram intra-pelvic accessory genital glands; seminal vesicles (SV), Cowper's glands (CG), and prostate gland (PG) in relation to the urinary bladder (UB; non-echogenic) and the bulbospongiosus muscle (BSM) and urethral muscle (UM). Note that the urethra appears as lumen with different echogenicity inside the prostate gland **(B)**.

The testes, epididymis and accessory genital glands were examined ultrasonography (Hitachi, EUB-405B, Japan) on D10, D20, and D30. The testes and epididymis was scanned using 3.5 MHz convex array transabdominal probe. While, the accessory genital glands, such as the seminal vesicles (SV), prostate gland (PG), and bulbourethral or Cowper's glands (CG), were scanned per rectum using 5/7.5 MHz linear array transrectal probe. The transducer was fitted with a self-manufactured connector to favor its manipulation per rectum. The measurement of the testicular width (TW), testicular depth (TD), the tail of the epididymis (TE), and the intra-pelvic accessory genital glands, such as SV, CG at both right and left sides, and the PG were recorded for each ram on both sides (right and left), as well (Figure 2). All examinations were done by the same operator to avoid individual variation.

### Semen Collection and Evaluation

The sexual behavior for each ram was observed daily (8:00–9:00 am). The pen-libido test was evaluated by introducing rams to teaser ewes. All experimental rams were left to move out of the collection area without being restrained to observe their sexual activity. The one false mount was performed by all rams before each semen collection. Semen samples had been collected using an artificial vagina (2 times weekly) starting from D14 until D60. Semen was immediately evaluated for gross (volume, color, and pH) and microscopic (Nikon E 400) (mass motility, concentration, viability, and abnormalities) parameters. Semen volume (ml) was measured using a graduated collection tube. Initial pH of semen samples was measured by comparative nitrating pH papers. Mass motility was assessed at a low magnification (10×), scored on a scale from 0 (no motility) to 5 (excellent motility). The individual motility was given a score in percentages after dilution with sodium citrate buffer solution (2.9%) with ration 4:1 (buffer: semen). For evaluation of the viability of spermatozoa, fixed-smears were stained with Eosin (Carl Roth Gmbh+Co. KG, Karlsruhe, Germany)–Nigrosine (Sigma-Aldrich, Saint Louis, MO, USA) stain and examined at a 400× magnification using. A total of 300 spermatozoa was examined where colored/stained head spermatozoa were calculated as dead, and unstained ones as viable. Primary and secondary abnormalities of spermatozoa were examined. Spermatozoa concentration was determined using hemocytometer. And the total number of spermatozoa per ejaculate was calculated by multiplying the ejaculate volume × concentration per ml. Reaction time (in seconds) was recorded using stopwatch as the time required for mounting teaser ewe until complete ejaculation (18).

### Blood Sampling

Blood samples were collected from the jugular vein by venipuncture into plain and heparinized collecting tubes at D10, D20, and D30. Blood samples centrifuged at 4,000 rpm for 15 minutes, then sera and plasma samples were harvested and stored at−20°C until further assays. Collecting tubes coated with sodium fluoride were used for determination of glucose levels. Plasma glucose and urea were determined according to Caraway and Watts using assay kits supplied by the Diamond Chemical Company, Germany (19). Aspartate transaminase (AST) and alanine transaminase (ALT) were determined according to Young using assay kits supplied by Spectrum Chemical Company, Egypt (20). The serum triiodothyronine (T3) and thyroxine (T4) concentrations (ug/dl), were assayed using ELISA kits (Atlas Medical Co, CB4, 0WX, United Kingdom) (UK). Serum glutathione peroxidase level was measured using photometer and commercial test kits (Biodiagnostic Co., Egypt). The glutathione peroxidase (GPX) activity was measured according to the method of Paglia and Valentine (21), which is based on the oxidation of reduced glutathione (GSH) to oxidized glutathione (GSSG) catalyzed by GPX. GPX activity was measured based on NADPH consumption in the enzyme-coupled reactions through the standard equation. While, Serum testosterone level was measured using ELISA kits (Bio Check, Foster City, CA 94404, USA). All measurements were done according to manufacturer protocols provided in the kits.

### Statistical Analysis

The results were expressed as means ± standard errors of the means (SEM). Statistical analysis of the presented data was performed by using the SPSS computer programs (2007). The independent *t*-test was used to compare between the two groups and to show the effect of rumen-protected L-arginine oral supplementation. The normal distribution of the data was confirmed using the Kolmogorov–Smirnov test. The *p* values ≤ 0.05 were considered significant.

## Results

### Ultrasonography of the Tests and Accessory Genital Glands in Rumen-Protected L-Arginine Supplemented Group and Control Group

The ultrasonographic biometric measurements of testicular, epididymal, and accessory genital glands in control, and L-arginine treated groups are presented in Table 1.

**Table 1:** Ultrasonographic biometric measurements of the testes and accessory genital glands (both sides) in the control and L-arginine treated ram at days 10, 20, and 30 (*n* = 5/group, mean ± SEM).

**Parameters**	**Day**		**Control**	**L-arginine**
Testicular width	D10	Right	3.75 ± 0.28	3.89 ± 0.17
		Left	3.75 ± 0.25	3.87 ± 0.16
	D20	Right	3.48 ± 0.24	4.37 ± 0.36*
		Left	2.93 ± 0.39	4.31 ± 0.26*
	D30	Right	3.04 ± 0.37	4.59 ± 0.13**
		Left	2.97 ± 0.44	4.24 ± 0.15*
Testicular depth	D10	Right	3.86 ± 0.23	3.63 ± 0.26
		Left	3.63 ± 0.31	3.74 ± 0.40
	D20	Right	3.24 ± 0.25	4.31 ± 0.28*
		Left	2.99 ± 0.41	4.15 ± 0.37*
	D30	Right	3.12 ± 0.33	4.56 ± 0.15**
		Left	3.27 ± 0.37	4.34 ± 0.35
Tail of the epididymis	D10	Right	1.61 ± 0.14	1.64 ± 0.15
		Left	1.62 ± 0.09	1.76 ± 0.17
	D20	Right	1.72 ± 0.11	2.43 ± 0.12**
		Left	1.60 ± 0.21	2.46 ± 0.23*
	D30	Right	1.97 ± 0.33	2.92 ± 0.25*
		Left	2.32 ± 0.22	2.85 ± 0.19
Seminal vesicle	D10	Right	1.04 ± 0.04	1.39 ± 0.13*
		Left	0.98 ± 0.07	1.20 ± 0.07
	D20	Right	1.04 ± 0.07	1.37 ± 0.08*
		Left	0.94 ± 0.06	1.44 ± 0.17*
	D30	Right	1.28 ± 0.15	1.79 ± 0.11*
		Left	1.05 ± 0.20	1.57 ± 0.05*
Cowper's Gland	D10	Right	1.24 ± 0.09	1.55 ± 0.04*
		Left	1.06 ± 0.08	1.19 ± 0.03
	D20	Right	1.36 ± 0.14	1.53 ± 0.13
		Left	1.19 ± 0.13	1.45 ± 0.17
	D30	Right	1.17 ± 0.06	1.81 ± 0.24*
		Left	1.10 ± 0.12	1.29 ± 0.09
Prostate gland	D10		0.86 ± 0.11	0.98 ± 0.11
	D20		0.92 ± 0.12	1.02 ± 0.15
	D30		0.76 ± 0.09	0.99 ± 0.05

*Means in the same row differ significantly (p ≤ 0.05); **Means in the same row differ significantly (p ≤ 0.01).

Our results revealed that at D10, the ultrasound biometric measurements of the RSV and RCG (mean ± SEM) were significantly higher in the L-arginine treated (1.39 ± 0.13 and 1.55 ± 0.04) in comparison to the Control group (1.04 ± 0.04 and 1.24 ± 0.09) for the right side seminal vesicle (RSV) and right side Cowper's gland (RCG), respectively. No significant differences (*P* > 0.05) were found in testicular width of the right and left testes (RTW, and LTW), testicular depth of the right and left testes (RTD, and LTD), tail of the epididymis at the right and left sides (RTE, and LTE), left side seminal vesicle (LSV), left side Cowper's gland (LCG), and PG between L-arginine and Control group (Table 1).

At D20, all testicular and most of accessory genital glands measurements were significantly higher (*p* < 0.05) in the L-arginine treated group in comparison to Control group with values (mean ± SEM) of 4.37 ± 0.36, 4.31 ± 0.28, 2.43 ± 0.12, 1.37 ± 0.08, 4.31 ± 0.26, 4.15 ± 0.37, 2.46 ± 0.23, and 1.44 ± 0.17 in the L-arginine treated group and 3.48 ± 0.24, 3.24 ± 0.25, 1.72 ± 0.11, 1.04 ± 0.07, 2.93 ± 0.39, 2.99 ± 0.41, 1.60 ± 0.21, and 0.94 ± 0.06 in the Control group for the RTW, RTD, RTE, RSV, LTW, LTD, LTE, and LSV, respectively. While there were no significant differences (*P* > 0.05) in the RCG, LCG, and PG measurements between L-arginine treated and Control groups (Table 1).

At D30, most of testicular and accessory genital glands measurements were significantly higher (*p* < 0.05) in the L-arginine treated group in comparison to Control group with values (mean ± SEM) of 4.59 ± 0.13, 4.56 ± 0.15, 2.92 ± 0.25, 1.79 ± 0.11, 1.81 ± 0.24, 4.24 ± 0.15, 1.57 ± 0.05 in the L-arginine treated and 3.04 ± 0.37, 3.12 ± 0.33, 1.97 ± 0.33, 1.28 ± 0.15, 1.17 ± 0.06, 2.97 ± 0.44, 1.05 ± 0.20 in the Control group for the RTW, RTD, RTE, RSV, RCG, LTW, and LSV, respectively. While, no significant differences (*p* > 0.05) were observed in the LTD, LTE, LCG, and PG between L-arginine and Control groups (Table 1).

### Sexual Behavior, Semen Evaluation, and Serum Testosterone Concentrations in Rumen-Protected L-Arginine Supplemented Group and Control Group

The results of pen-libido test (estimated with reaction time), the measured semen parameters and testosterone concentration in Control and L-arginine treated groups are presented in Table 2.

**Table 2:** Reaction time, semen parameters and testosterone concentrations in the control and L-arginine treated rams (*n* = 5/group, mean ± SEM).

**Parameter**	**Control**	**L-arginine**
Reaction time (s)	35.50 ± 6.30	8.90 ± 2.70***
Ejaculate volume (ml)	0.83 ± 0.03	1.17 ± 0.03**
pH	7.14 ± 0.02	6.66 ± 0.0***
Mass motility (score)	3.50 ± 0.19	4.20 ± 0.17**
Individual motility (%)	76.90 ± 0.57	85.20 ± 0.63***
Sperm cell conc. (x10^9^/ml)	1.57 ± 0.04	2.96 ± 0.03***
Alive spermatozoa (%)	63.70 ± 1.98	78.50 ± 2.01***
Total sperm abnormalities (%)	29.94 ± 0.93	17.80 ± 0.56***
Serum testosterone (ng/ml)	1.13 ± 0.03	1.35 ± 0.04***

**Means in the same row differ significantly (p ≤ 0.01); ***Means in the same row differ significantly (p ≤ 0.001).

For the pen-libido test, the reaction time (seconds; mean ± SEM) was significantly shorter (*P* < 0.05) in the L-arginine treated group (8.9 ± 2.7) than that in the Control group (35.5 ± 6.3) (Table 2).

For semen evaluation parameters, there were significant improvements in all the measured parameters in the L-arginine treated group in comparison to those in the Control group; the ejaculate volume (ml; mean ± SEM) was significantly larger (*p* < 0.05) in the L-arginine treated group (1.17 ± 0.03) than that in the Control group (0.83 ± 0.03), the pH value (mean ± SEM) was significantly lower (*p* < 0.05) in the L-arginine treated group (6.66 ± 0.00) than that in the Control group (7.14 ± 0.02), the mass motility (score 1–5; mean ± SEM) was significantly higher (*p* < 0.05) in the L-arginine treated group (4.2 ± 0.17) than that in the Control group (3.5 ± 0.19) and the individual motility (%; mean ± SEM) was significantly higher (*p* < 0.05) in the L-arginine treated group (85.2 ± 0.63) than that in the Control group (76.9 ± 0.57), the concentration of spermatozoa (x109/ml; mean ± SEM) was significantly higher (*p* < 0.05) in the L-arginine treated group (2.96 ± 0.03) than that in the Control group (1.57 ± 0.04), percentage of alive spermatozoa (%; mean ± SEM) was significantly higher (*p* < 0.05) in the L-arginine treated group (78.5 ± 2.01) than that in the Control group (63.7 ± 1.98), and the percentage of total sperm abnormalities (%; mean ± SEM) was significantly lower (*p* < 0.05) in the L-arginine treated group (17.8 ± 0.56) than that in the Control group (29.94 ± 0.93) (Table 2).

For the testosterone, serum testosterone level (ng/ml; mean ± SEM) was significantly higher (*p* < 0.05) in the L-arginine treated group (1.35 ± 0.04) than that in Control group (1.13 ± 0.03) (Table 2).

### Serum Biochemical Parameters and Glutathione Peroxidation Activities in Rumen-Protected L-Arginine Supplemented Group and Control Group

The results of the measured biochemical parameters and glutathione peroxidation activities in Control and L-arginine treated groups are presented in Table 3.

**Table 3:** Serum biochemical parameters and glutathione peroxidation levels rams in the control and L-arginine treated rams at days 10, 20, and 30 (*n* = 5/group, mean ± SEM).

**Parameter**	**Day**	**Control**	**L-arginine**
Glucose (mg/dl)	D10	59.03 ± 1.70	69.47 ± 6.97
	D20	67.50 ± 0.79	72.32 ± 3.28
	D30	70.71 ± 1.98	86.89 ± 1.49**
Cholesterol (mg/dl)	D10	67.62 ± 1.40	66.32 ± 1.24
	D20	66.73 ± 0.59	76.32 ± 0.53**
	D30	67.17 ± 0.79	76.98 ± 0.83**
Urea (mg/dl)	D10	40.86 ± 0.89	44.04 ± 1.61
	D20	42.04 ± 3.02	50.66 ± 2.94
	D30	44.35 ± 1.50	56.97 ± 0.45**
Aspartate transaminase (AST; mg/dl)	D10	43.33 ± 3.84	43.00 ± 2.30
	D20	42.33 ± 1.66	42.25 ± 0.47
	D30	44.00 ± 3.21	47.00 ± 1.08**
Alanine transaminase (ALT; mg/dl)	D10	25.06 ± 1.56	25.05 ± 1.24
	D20	26.42 ± 1.28	32.00 ± 2.34
	D30	27.02 ± 1.66	34.00 ± 2.30
Triiodothyronine (T3; ng/dl)	D10	0.13 ± 0.01	0.12 ± 0.01
	D20	0.18 ± 0.01	0.21 ± 0.03
	D30	0.15 ± 0.01	0.18 ± 0.01
Thyroxine (T4, ng/dl)	D10	0.20 ± 0.08	0.21 ± 0.01
	D20	0.19 ± 0.02	0.30 ± 0.03
	D30	0.22 ± 0.00	0.32 ± 0.03*
Glutathione peroxidase (GSH-Px; U/ml)	D10	119.5 ± 10.3	258.2 ± 9.7***
	D20	117.1 ± 12.3	271.3 ± 11.4***
	D30	112.4 ± 9.4	264.6 ± 8.5***

*Means in the same row differ significantly (p ≤ 0.05); **Means in the same row differ significantly (p ≤ 0.01); ***Means in the same row differ significantly (p ≤ 0.001).

For the energy related parameters, at D10, there were no significant changes in the glucose levels (mg/dl) and cholesterol levels (mg/dl) between L-arginine treated and Control group. At D20, there were no significant differences in the glucose levels (mg/dl) between L-arginine treated and Control group, while the cholesterol level (mg/dl; mean ± SEM) was significantly higher (*p* < 0.05) in the L-arginine treated group (76.32 ± 0.53) than that in Control group (66.73 ± 0.59). At D30, glucose (mg/dl; mean ± SEM) and cholesterol levels (mg/dl; mean ± SEM) were significantly higher in the L-arginine treated in comparison to those in the Control group (Table 3).

For urea levels (mg/dl), no significant differences were found in the urea levels (mg/dl) between L-arginine treated and Control group at D10, and D20. While, at D30, the urea level (mg/dl; mean ± SEM) was significantly higher in the L-arginine treated (56.97 ± 0.45) in comparison to those in the Control group (44.35 ± 1.50) (Table 3).

For liver function enzymes, no significant differences were reported in the AST (mg/dl) and ALT levels (mg/dl) between L-arginine treated and Control group at D10, and D20. While, at D30, the AST level (mg/dl; mean ± SEM) was significantly higher in the L-arginine treated (47.00 ± 1.08) in comparison to those in the Control group (44.00 ± 3.21) with no significant difference in the ALT levels (mg/dl) between L-arginine treated and Control group (Table 3).

For THs, no significant differences were reported in the T3 (ng/dl) and T4 levels (ng/dl) between L-arginine treated and Control group at D10, and D20. While, at D30, the T4 level (ng/dl; mean ± SEM) was significantly higher in the L-arginine treated (0.32 ± 0.03) in comparison to those in the Control group (0.22 ± 0.00) with no significant difference in the T3 levels (mg/dl) between L-arginine treated and Control group (Table 3).

For glutathione peroxidation activities (GSH-Px), serum GSH-Px levels (U/ml; mean ± SEM) were significant higher (*p* < 0.05) in the L-arginine treated than those in Control group, at all time-points, with values (mean ± SEM) of 258.2 ± 9.7, 271.3 ± 11.4, and 264.6 ± 8.5 in the L-arginine treated group and 119.5 ± 10.3, 117.1 ± 12.3, and 112.4 ± 9.4 at D10, D20, and D30, respectively (Table 3).

## Discussion

Our study successfully evaluated the effect of oral supplementation of rumen-protected L-arginine on rams' fertility. The oral route is an economical, easily applied, almost safe in different animal species (22). Moreover, in this study, the rumen-protected form of L-arginine was prepared as pellets, which enable the animals to voluntarily consume the rumen-protected L-arg.

The findings in the present study reported that oral supplementation of rumen-protected L-arginine markedly improved the rams' fertility. It improved the ultrasonographic biometric measures of the testes, TE, SV, and CG. Moreover, the oral supplementation of rumen-protected L-arginine enhanced the ram's sexual behavior, semen quality and quantity. In addition, the biochemical measurements ensure safety and efficacy of the oral administration of rumen-protected L-arginine in rams.

The improvement in the ultrasonographic biometric measurements reported in the rumen-protected L-arginine treated group is likely because of the notion that L-arginine increases the synthesis of NO (2, 23), thus, increasing blood flow to testes and accessory genital glands which in turn, leads to improving the performance and function of these reproductive organs (5), and reflected by the increase in the ultrasonographic biometry.

The marked enhancement in the rams' semen characters (quantity and quality) after supplementation with rumen-protected L-arginine reported in the present study, in addition, the marked reduction in the reaction time as the pen libido test could be also referred to as the increase in NO level which plays an important role in the arginase enzyme activity as previously reported (23). The current reported findings are supported by the recent findings recorded by Kaya et al. who evaluated the effect of intraperitoneal administration of L-arginine on spermatological parameters in rams (13). The NO plays an important role in vasodilatation (24), and it is well-known that, NO increases blood flow to most organs, such as the reproductive organs. Moreover, Yasuda suggested that the increased NO concentrations is likely due to the increase in cGMP release (6) which increases the testicular blood flow, and in turn, increases the transport of nutrients and hormones to reproductive organs (25), such as the accessory genital glands.

The increase in the semen volume reported in the present study in the rumen-protected L-arginine supplemented group compared to the control group is likely due to the marked increase in the biometric parameters of the testes, epididymis, SV and CG reported in this study. Our findings are supported by those recently found by Kaya et al. who reported significant increase in the semen volume in the intraperitoneally injected L-arginine treated group (13). In contrast to our findings and the recent study in rams by Kaya et al. and Wu et al. found that 1% L-arg-HCl included in the diets for 30 days has no effect on the amount of semen in pigs, that could referred to species difference, the form of L-arg, and/or the dose difference (13, 15).

The higher mass activity and the individual motility of spermatozoa reported in the present studyin the L-arginine group compared to the control 1 is likely due to the polyamine-mediated effects of the L-arginine that, the ornithine formed by arginase enzyme in polyamine biosynthesis is converted to putrescine, then putrescine is involved in the biosynthesis of spermine and spermidine (26). This present finding is supported by Ratnasooriya and Dharmasiri findings reported that L-arginine orally administered to rats resulted in a marked increase in motility of the epididymal spermatozoa (27).

The present study revealed significant increase in the concentrations and viability of spermatozoa in the L-arginine treated group compared to the control group, these results coincided with those previously reported in human (28, 29), rams (13), and buck rabbits (30). It should be noted that, a close relationship between testicular size and production of spermatozoa was previously reported, more specifically, rams with smaller testes may not produce enough spermatozoa through the mating period (31). The positive effect of L-arginine on the spermatozoa vitality is supported by the increased in the glucose level in L-arginine treated group reported in this study. Moreover, Scibona et al. reported that oral administration of L-arginine increase concentration of spermatozoa without causing any side effects in men (14). In our study, the lower percentage of abnormal spermatozoa in the L-arginine treated group is supported by the previous findings suggested that dietary L-arginine deficiency caused abnormalities in the spermatozoa (32).

In the current study, the higher testosterone level in the L-arginine treated group is more possibly due to the NO-increasing effect on the testicular blood flow.

Interestingly, our study found significant increase in the glucose levels after 1 month of L-arginine oral administration. As known, glucose uptake and metabolism by testicular tissue have an essential role in the male reproductive performance, moreover, normal glucose metabolism is critical for spermatogenesis since the developing germ cells in the Sertoli cells consume lactate, a waste product of glucose, as their main energy source (8).

Higher urea levels reported in this study in the L-arginine treated rams after 30 days is likely due to conversion of L-arginine into L-ornithine and urea (33). Similar AST and ALT activity reported in both L-arginine treated group and control groups ensure the safety of the oral administration of L-arginine in rams. However higher T4 level reported at D30 in the L-arginine treated group is beneficial to improve different cellular physiological processes involving adenosine tri-phosphate utilization (34). While, the significantly higher serum GSH-Px in the L-arginine treated group prove the positive protective effect of the L-arginine in rams as GSH has pro-oxidant effect in addition to its antioxidant role (35).

It should be noted that, the findings of the present study in regard to the efficacy of the oral administration of the rumen-protected L-arginine supported the notion that the pharmacokinetics of substances administered by the oral route used in our study or intraperitoneally as recently reported by Kaya et al. are more similar (13), because the absorption in both administration routs is into the portal vein through mesenteric vessels (36). However, oral administration is considered as a safe, convenient, and economic route of administration.

## Conclusion

In conclusion, treatment with rumen-protected L-arginine is beneficial in improvement of rams' fertility that it could improve the testicular, accessory genital glands biometric measurements and functions, and semen quality and quantity. Moreover, the biochemical findings measured in the present study ensured the efficacy and safety of the oral administration of rumen protected L-arginine in rams. Further studies are needed to evaluate the impact of different concentrations of Rumen-Protected L-Arginine Oral Supplementation on ram fertility.

## Data Availability Statement

The raw data supporting the conclusions of this article will be made available by the authors, without undue reservation.

## Ethics Statement

The animal study was reviewed and approved by Egyptian Medical Research Ethics Committee (No. 14-126).

## Author Contributions

HH, MA, AMA, SF, and AEA prepared conception and design of study, performed data curation, and interpretation of data. HH and SF conducted the field study, blood sampling and ultrasonographic examination, collected laboratory samples, and conducted biochemical analyses. MA and AMA manipulated and statistically analyzed the data. AEA, AH, and MA drafted the manuscript. RS, AS, and AEA carried out final writing, critical review, and revision. All authors have read and approved the final manuscript.

## Conflict of Interest

The authors declare that the research was conducted in the absence of any commercial or financial relationships that could be construed as a potential conflict of interest.

## Publisher's Note

All claims expressed in this article are solely those of the authors and do not necessarily represent those of their affiliated organizations, or those of the publisher, the editors and the reviewers. Any product that may be evaluated in this article, or claim that may be made by its manufacturer, is not guaranteed or endorsed by the publisher.
